# Mapping the Interaction Anatomy of BmP02 on Kv1.3 Channel

**DOI:** 10.1038/srep29431

**Published:** 2016-07-11

**Authors:** B. Wu, B. F. Wu, Y. J. Feng, J. Tao, Y. H. Ji

**Affiliations:** 1 Lab of Neuropharmacology and Neurotoxicology, Shanghai University, Nanchen Road 333, Shanghai 200444, China; 2 Central Laboratory, Putuo Hospital, Shanghai University of Traditional Chinese Medicine, 164 Lanxi road, Shanghai 200062, China

## Abstract

The potassium channel Kv 1.3 plays a vital part in the activation of T lymphocytes and is an attractive pharmacological target for autoimmune diseases. BmP02, a 28-residue peptide isolated from Chinese scorpion (*Buthus martensi Karsch)* venom, is a potent and selective Kv1.3 channel blocker. However, the mechanism through which BmP02 recognizes and inhibits the Kv1.3 channel is still unclear. In the present study, a complex molecular model of Kv1.3-BmP02 was developed by docking analysis and molecular dynamics simulations. From these simulations, it appears the large β-turn (residues 10–16) of BmP02 might be the binding interface with Kv 1.3. These results were confirmed by scanning alanine mutagenesis of BmP02, which identified His9, Lys11 and Lys13, which lie within BmP02’s β-turn, as key residues for interacting with Kv1.3. Based on these results and molecular modeling, two negatively charged residues of Kv1.3, D421 and D422, located in turret region, were predicted to act as the binding site for BmP02. Mutation of these residues reduced sensitivity of Kv 1.3 to BmP02 inhibition, suggesting that electrostatic interactions play a crucial role in Kv1.3-BmP02 interaction. This study revealed the molecular basis of Kv 1.3 recognition by BmP02 venom, and provides a novel interaction model for Kv channel-specific blocker complex, which may help guide future drug-design for Kv1.3-related channelopathies.

Kv1.3 is a voltage-gated potassium channel that is crucial for T cell development and activation. Kv1.3 is highly expressed in effector memory T (T_EM_) cells, and constitutes the predominant K^+^ conductance for these cells, and provides the electrochemical driving force for Ca^2+^ influx which is necessary for both T-cell proliferation and cytokine secretion^1^,^2^. Thus, Kv1.3 is regarded as an attractive target for treatment of autoimmune diseases^3^,^4^. To date, several Kv1.3 channel blockers, such as 5-methoxypsoralen and acacetin have been proven to suppress cytokine secretion of T cells and alleviated autoimmune diseases in animal or cell models^5^,^6^.

α-KTxs are the most well studied and abundant short-chain toxins in scorpion venom^7^. Members of this toxin-family are usually short peptides, ranging from 28–42 amino acids long. Most of them share a cysteine-stabilized α-helical and β-sheet (CSαβ) fold, composed of a single α-helix and two or three folds held together by three or four disulfide bridges^8^. α-KTxs block potassium channels through a highly conserved dyad motif centered on a lysine located in the β strand, which was first revealed in the crystal structure of a complex between Charybdotoxin from scorpion *Leiurus quinquestriatus* and the Kv1.2–2.1 paddle chimera channel^9^,^10^. This dyad-mediated blockade is the most common interaction-mode between scorpion toxins and Kv channels, and is also a inhibitory mechanism commonly observed in other natural toxins from other species, such as sea anemones, snakes and cone snails^11^,^12^,^13^,^14^.

BmP02, also referred to α-KTx9.1, is a peptide toxin venom from the Chinese scorpion (*Buthus martensi* Karsch): it is comprised of 28 amino acids, whose tertiary structure is stabilized by 3 disulfide bonds^15^,^16^. BmP02 can inhibit transient outward potassium currents in rat ventricular myocytes, and weakly block SK channels in the rat brain^17^,^18^. BmP02 showed high sequence identity with BmP03 and Kbot1, however, unlike Charybdotoxin and other α-KTxs, BmP02 does not contain a dyad motif^16^,^19^. Despite lacking a dyad motif, BmP02 is a potent current blocker of the Kv1.3 channel^20^. It is still unclear how BmP02 is able to recognize and block Kv1.3 channels. In this study, we employ whole cell patch clamping, molecular dynamic simulations, and scanning mutagenesis to elucidate the interaction between synthetic BmP02 and Kv1.3 channel expressed in a HEK293T cell line, to better understand the structure-function relationship between the venom and the potassium channel, and the interaction mode between dyad free toxins and Kv channels.

## Results

### Selective blockade of sBmP02 on Kv1.3

**Figure 1:**
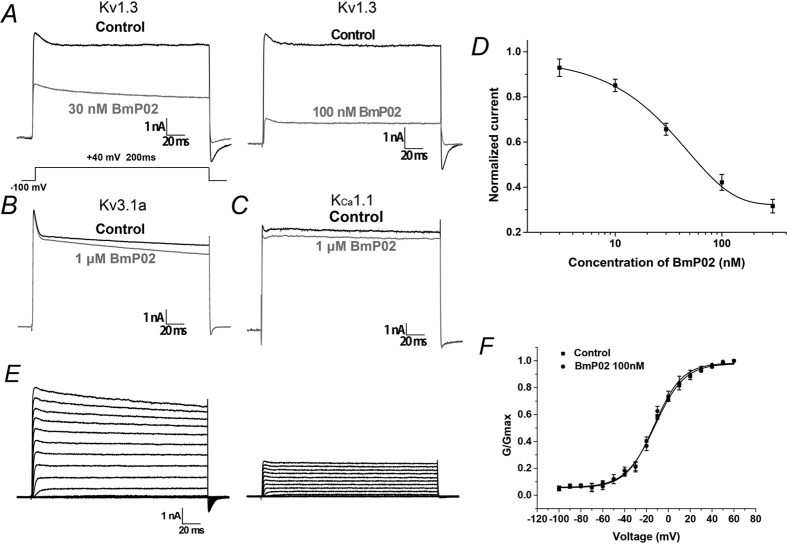
Pharmacological effect of sBmP02 on potassium channels. (**A**) Current responses of Kv1.3 channels to a test voltage of +40 mV in the presence of vehicle (black trace) or presence of 30 nM and 100 nM sBmP02, respectively (in gray trace). (**B,C**) Current responses of Kv3.1a and KCa1.1 channels to a test voltage of +40 mV in the presence of vehicle (black trace) or presence of 1 μM sBmP02 (in gray trace). (**D**) The dose-response curve of sBmP02 inhibiting Kv1.3 currents was fitted by the Hill equation. The EC50 is 32.1 ± 2.7 nM. n = 10, mean ± SEM. (**E**) Representative traces of K+ currents in blank (left) and 100 nM sBmP02 (right) elicited by depolarizing pulses from a holding potential of −100 mV to +60 mV in 10 mV increments. (**F**) Normalized G-V relation of Kv1.3 in the absent (squares) and presence (dots) of 100 nM sBmP02. n = 10; mean ± SEM.

To determine whether our synthesized BmP02 (sBmP02) was capable of inhibiting Kv1.3 channels, HEK293T cells expressing the Kv1.3 channels were treated with increasing concentrations of sBmP02. 100 nM sBmP02 exhibited significant inhibition of Kv1.3 channel conductance, decreasing it by 50% during the depolarization at +40 mV (I_f_ = 0.42 ± 0.03, n = 10). At higher concentrations (1 μM), sBmP02 inhibition of Kv3.1a and KCa1 was decreased (Fig. 1B,C). The EC_50_ value was determined to be 32.10 ± 2.67 nM (Fig. 1D). The results demonstrated that the inhibitory effects of sBmP02 on Kv1.3 mimic the effects of the naturally derived toxin.

To measure whether sBmP02 would modify the voltage dependence of Kv1.3 channel gating, the currents were elicited by depolarizing pulses from a holding potential of −100 mV to +60 mV in 10 mV increments (Fig. 1E). The steady-state activation curves determined by depolarization ranging from −100 to +60 mV did not shift significantly after a 10-min exposure to 100 nM sBmP02 (−12.2 ± 0.8 mV compared to −12.9 ± 1.0 mV, n = 10, P > 0.05, Fig. 1F). The voltage-independent modulation of sBmP02 suggested that BmP02 does not affect the voltage sensor of Kv1.3.

### Simulating the action mode of BmP02 and Kv1.3

Since sBmP02 did not affect voltage sensing by Kv1.3, it was hypothesized that the pore region of Kv1.3 interacted with sBmP02. A homology model of Kv1.3 pore region was constructed using the X-ray crystal structures of the KcsA channel as the template^21^, which bears sequence homology (52.1%) to Kv1.3 (Figure S5A,B). The predicted pKa distribution on the surface of Kv1.3 channel and BmP02 are shown in Figure S1D. The vestibule of Kv1.3, which lies on the outside of the cell membrane is negative charged, whereas positive charged residues of BmP02 are mainly distributed in the C terminal of the α helix (His9) and the large β turn (residues 10–16). Remarkably, three basic residues His9, Lys11 and Lys13 are clustered closely together, and their highly solvent-exposed side chains are positioned to potentially coordinate docking with a receptor.

**Figure 2:**
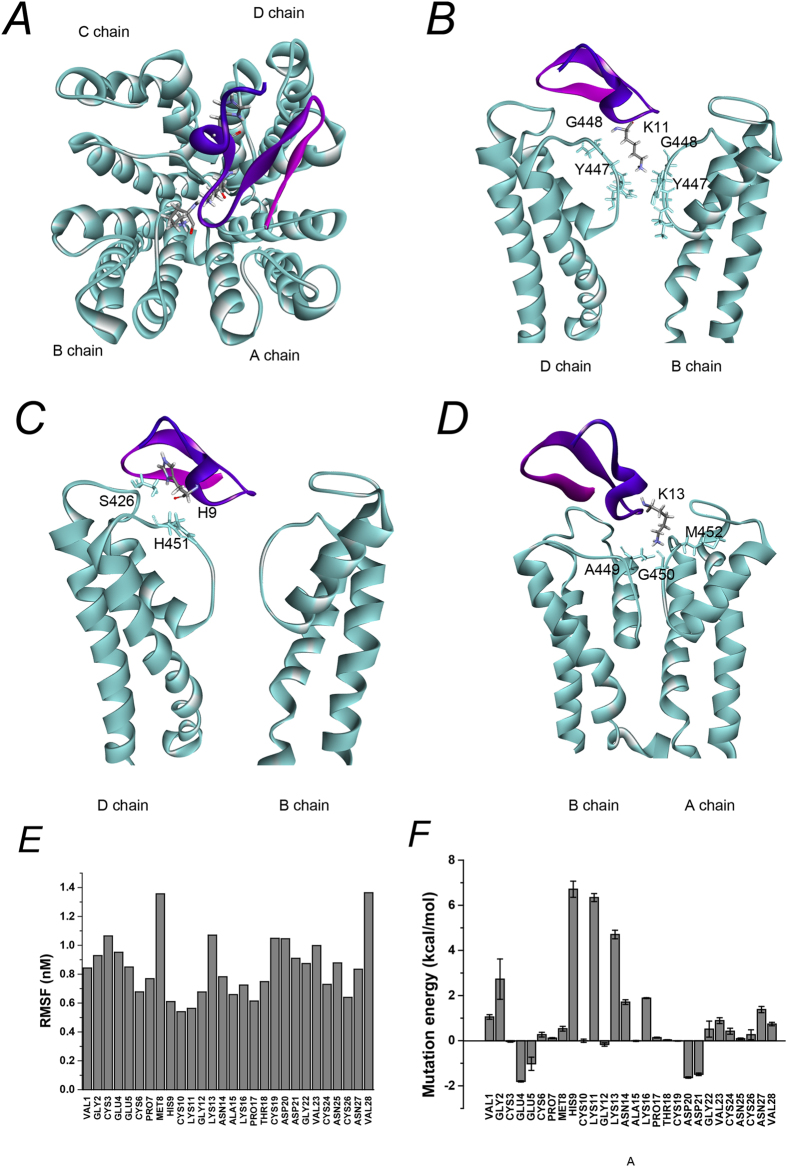
Modeling of BmP02 interactions with Kv1.3 channels. (**A**) Interaction mode of BmP02 with Kv1.3 channel. BmP02 key functional residues are marked. (**B**) Kv1.3 channel pore-blocking Lys11 of Bmp02 primarily contacts the conserved residues of Kv1.3 channel pore region within a contact distance of 5 nm. (**C**) His9 of BmP02 interacts with Ser426 and His451 in channel D chain within a contact distance of 5 nm. (**D**) Lys13 of BmP02 mainly contacted channel Asp449 in B chian, Gly450 and Met 452 in A chain within a contact distance of 5 nm. (**E**) The RSMF value of all BmP02 residues during the MD simulation. (**F**) Mean values of Virtual Alanine Mutation of Kv1.3-BmP02 complex obtained from ZDOCK and MD simulations with standard deviation.

The channel-toxin complex was obtained by protein-protein docking. A more native-like complex structure was established using a 10 ns molecular dynamic simulation. The conformations were stable after 5 ns simulation (Figure S6). As shown in the model, BmP02 projects a lysine (Lys11) into the pore of the channel, and forms hydrogen bonds with several residues in the selective filter (Fig. 2B and Table 1). Hydrogen bond interactions are localized in the binding interface. Residues His9, Lys11 and Lys13 appeared to be the key contacts between the vestibule of Kv1.3 channel and BmP02, via 15 hydrogen bonds (Fig. 2B–D and Table 1).

During the 10 ns MD simulation, the RMSF of BmP02 residues was limited, especially in the large β turn and adjacent regions (no more than 0.8 in residues 9–12 and 14–18, Fig. 2E), indicating that this region was stable in all conformations during the MD simulation. To determine whether the residues of BmP02 involved in the interaction between the toxin and the channel, Virtual Alanine Mutation Scanning was employed to calculate the energy changes when the residues of BmP02 were individually mutated to alanine. Mutation of His9, Lys11 and Lys13 generated the largest energy increments (6.71 ± 0.36 kcal/mol, 6.34 ± 0.18 kcal/mol, 4.71 ± 0.19 kcal/mol, respectively, Fig. 2F).

### The key residues of sBmP02 interacting with Kv1.3

**Figure 3:**
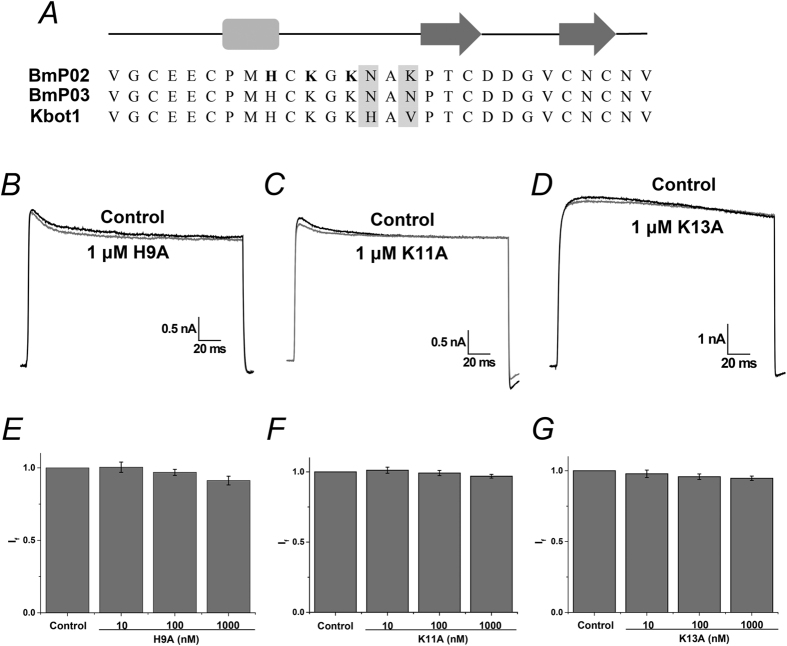
Determination of key residues for BmP02 contacts with Kv1.3. (**A**) Primary sequence of BmP02: the α helix are shown in square and the β strands in arrows. The key residues are shown in bold. (**B–D**) Representative currents of Kv1.3 in the absence (black) and presence (gray) of the mutant BmP02. (**E–G**) The fraction current of Kv1.3 in the presence of 10, 100, 1000 nM mutant BmP02, respectively.

To verify the simulated interaction mode between Kv1.3 and BmP02, three alanine point mutants of BmP02: H9A, K11A and K13A were synthesized and tested. Electrophysiological recording showed that the currents of Kv1.3 during the depolarization of +40 mV were not significantly suppressed after 10-min treatment of the cells with the three sBmP02 mutants (the fraction current was 91.2 ± 3.0%, 96.9 ± 1.3% or 94.7 ± 1.6% in the presence of 1 μM H9A, K11A or K13A, respectively, Fig. 3). The three sBmP02 mutants showed nearly identical structure when compared with wild type sBmP02 by ^1^H NMR and CD spectra (Figures S7 and S8).

### The key residues of Kv1.3 associating with sBmP02

**Figure 4:**
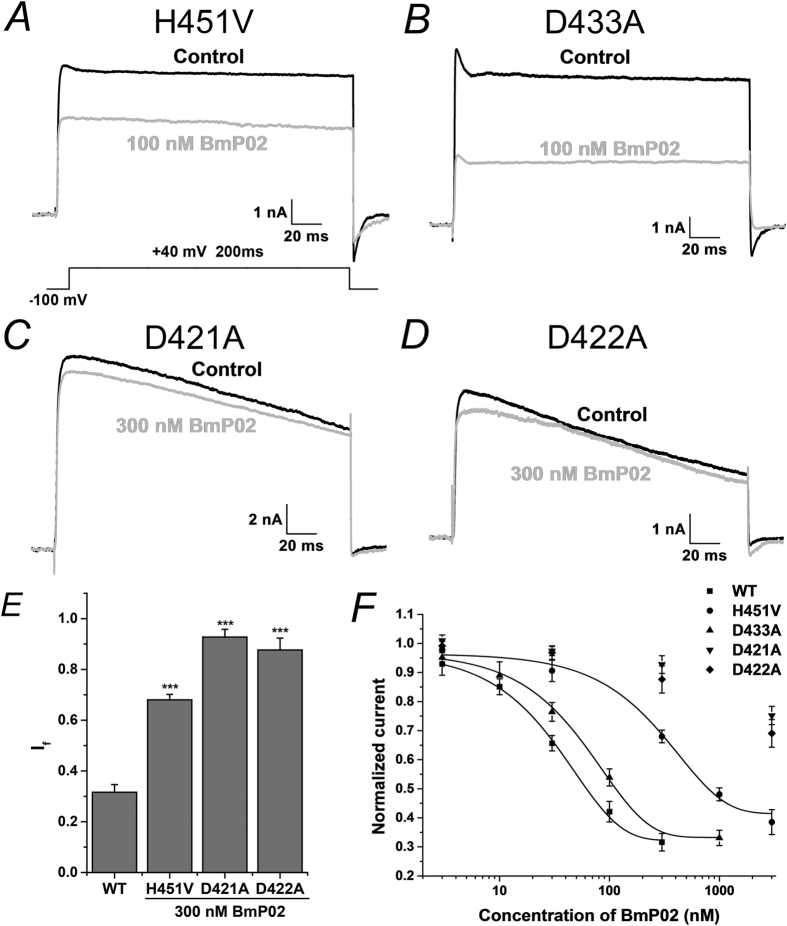
The charged residues of the Kv1.3 channel are involved in the recognition of sBmP02. (**A,B**) Current responses of mutant channels H451V and D433A, respectively, test voltage of +40 mV in the presence of vehicle control (in black trace) or in presence of 100 nM sBmP02. (**C,D**) Representative currents of mutant channels D421A and D422A in the absent (black) and present of 300 nM sBmP02. (**E**) The fraction current of WT Kv1.3 and mutant H451V, D421A and D422A at the presence of 300 nM sBmP02. (**F**) The dose-response relationship of sBmP02 inhibiting mutant channels H451V, D433A, D421A and D422, respectively.

The key residues of BmP02 are positively charged, which strongly suggested that negative charged residues on the external loops of Kv1.3 might play important roles in associating with BmP02. Four aspartic acid residues D449, D433, D421 and D422 located in the vestibule of Kv1.3 were individually mutated to alanine. No current was detected in the mutant D449A mutant during recording, possibly because this residue is vital in stabilizing the selectivity filter of Kv1.3. BmP02 showed weaker inhibition of the D433A mutant compared to the wild type channel (Fig. 4B). Mutation of D433 produced a 1.6-fold decrease in the apparent affinity for sBmP02 (the EC_50_ value increased to 52.55 ± 2.89 nM from 32.10 ± 2.67 nM, Fig. 4F). The mutants D421A and D422A showed little sensitivity to sBmP02 inhibition. Even after application of 300 nM sBmP02 for 10 minutes, the current readings of these two mutants were only reduced by 7.3 ± 3.0% and 11.4 ± 4.7%, respectively (n = 8–10, Fig. 4C–E).

The residue H451 at the BmP02 binding interface of Kv1.3 is replaced by a valine in the homologous channel Kv1.2. Mutation of H451 in Kv1.3 to valine decreased the channel’s affinity for sBmP02, with an EC_50_ value of 312.51 ± 36.44 nM, nearly a 10-fold increase compared with that of the wild-type channel (Fig. 4E,F).

## Discussion

α-KTxs are highly diverse family of toxins that contain two functional domains, both of which are capable of binding to different potassium channel subtypes. Some α-KTxs, such as P05, block SK channels using an RRCQ motif located in the α helical domain of the venom. Others, such as charybdotoxin, employ a dyad motif located in the β strands to bind to Shark-type channels and BK channels^9^,^22^. BmKTX has been demonstrated to interact with Kv1.3 by its turn motif although it also contains a dyad motif on its β-strand^21^,^22^. The interaction model between neurotoxin Pi1 and Kv1.2 showed that a special ring enriched basic residues in the toxin acts as the binding interface^23^. The novel action mode for α-KTxs provided in this study may also help to make better understanding of the interaction between KTxs and target channels.

BmP02 and two other toxins from α-KTx9 subfamily, BmP03 (from *Buthus martensi* Karsch) and Kbot1 (from *Buthus occitanus tunetanus*) are dyad free toxins, which have been demonstrated to be potent Kv1.3 blockers^16^,^19^,^20^. In this study the molecular interaction between BmP02 and Kv1.3 was explored to determine how BmP02 is acting on Kv1.3 in the absence of dyad motif. The Kv1.3-BmP02 complex model generated in by molecular simulation predicted that BmP02 utilizes a large β turn as the binding interface for Kv1.3, which was confirmed by site-directed mutagenesis (Figs 2 and 3). Electrostatic interactions between BmP02 and Kv1.3 were also shown to be essential for this interaction mode (Fig. 4).

Since the surface of the external vestibule of Kv1.3 is negative charged (Figure S2), it was hypothesized that the conserved positive charged residues K11 and K13 BmP02 might be important for interactions with Kv1.3. In the Kv1.3-BmP02 complex model established by protein-protein docking and molecular dynamic simulation, BmP02 employed the large β turn as the binding interface to act with Kv1.3. Virtual Alanine Mutation Scanning highlighted that His9, Lys11 and Lys13 within this β turn may be crucial for the interaction between BmP02 and Kv1.3. In charybdotoxin, Lys27 is the most critical residue and apparently projects into the pore of the channel^9^. Similarly, Lys11 in BmP02 was oriented to penetrate its side chain straight into the pore according to computer simulation although its structure is diverse to the classical dyad motif. It was proposed therefore that Lys11 might make hydrogen-bonding interactions with the backbone carbonyl oxygen of Tyr447 might disturb the coordination with a potassium ion at site S1. To test these hypotheses and validate the binding model, three BmP02 mutants H9A, K11A and K13A were geneterated. The overlapping curves of wild type BmP02 and the three mutants by ^1^H NMR and CD spectra indicated that they shared the same 3D architecture. Mutation of these residues abolished BmP02 mediated inhibition of Kv1.3 further indicating that this small motif composed by His9, Lys11 and Lys13 is crucial for BmP02 association with Kv1.3.

During dyad-mediated interactions of potassium channels and toxins, contacts between a conserved residue D433 located in the channel’s pore helix, and an Asn of the toxin on the binding interface are considered important^22^,^23^. However, in this study when D433 of BmP02 was mutated to alanine, the affinity for sBmP02 was reduced by only ~1.6 folds. This result lead to the supposition that D433 isn’t directly involved in the contact with BmP02, but that electrostatic forces between BmP02 and Kv1.3 were reduced. Interestingly, when two Asp residues (D421 and D422) located in the turret region of the channel were mutated, BmP02’s affinity for Kv1.3 was significantly reduced. Interestingly, these two residues seems to be relatively distant from the binding site of BmP02: Considering that the turret is the most outer region of the channel, the electrostatic potential on the surface may be dramatically changed by mutation of these Asp residues. One explanation for how this might affect BmP02 binding is that the electrostatic potential distribution on the surface of the channel may participate in the initial recognition of BmP02.

The results of the computer modeling indicated that D449 was likely an important residue in the channel-toxin interaction. Unfortunately, the D499A mutant did not respond in our electrophysiology assays. One possibility might be that D449 plays crucial roles in stabilizing the selective filter structure. However, from it was determined that the side chain of D449 in Kv1.3 interacts with H451. This is unique to Kv1.3: the equivalent Asp in Kv1.2 projects its side-chain in the opposite direction to the binding interface^24^,^25^. Therefore, mutation of H451 might mimic mutation of the D449 and disrupt binding to BmP02. Indeed, analysis of the H451V mutant showed a 10-fold increment in EC_50_, suggesting that D449 is indispensable in this interaction mode. In addition, the dominant force between BmP02 and Kv1.3 is hydrogen-bonding interactions, rather than canonical hydrophobic interactions observed in other dyad-mediated toxin-channel models. The hydrogen-bonding interaction between H451 of the channel and His9, Lys11 of BmP02 can partly explain why Kv1.2 showed less sensitivity to sBmP02.

Some natural Kv1.3 blockers have been proven to be specific drugs to autoimmune diseases. For instance, ADWX-1 is capable of alleviating experimental autoimmune encephalomyelitis in rats^26^. Compared to the classical α-KTxs, BmP02 uses a much more simple structure as the binding interface, which may provide a promising molecular template for peptide drug design, particularly in the treatment of autoimmune diseases.

## Materials and Methods

Plasmids carrying hKv1.3 (*kcna3*, NM_002232.4), KCa1.1 (*hSloα*, U23767) and Kv3.1a (*kcnc1a*, NM_001128725.1) were gifts from Yingliang Wu (Wuhan University), Noel Davies (University of Leicester) and Ping Song (Yale University), respectively. Point mutations were generated using sequential PCR using hKv1.3 channel as a template. The resulting mutants are as follows: D433A, D421A, D422A and H451V. Primers were designed by Primer 5.0 (PremierBiosoft, USA, Supplement Table 1).

All experiments were performed on HEK 293T cell lines. HEK 293T cells were obtained from Shanghai cell bank of Chinese Academy of Science. The cells were cultured in Dulbecco’s modified Eagle medium (DMEM; Life Technologies, Grand Island, NY) supplemented with 10% heat–inactivated fetal bovine serum (FBS; Gibco, Grand Island, NY). Culture dishes were incubated at 37° in a humidified atmosphere containing 5% CO_2_, and sub-cultured every 2~3 days. One day before transfection, HEK 293T cells were transferred to 24-well plates. At 90% confluence, cells were transiently transfected using Lipofectamine3000 (Invitrogen, USA) at a ratio of 1 μL reagent with 1 μg total plasmid per well. Electrophysiological experiments were performed 1~2 days after transfection.

### Electrophysiological recordings

Whole-cell voltage-clamp experiments were performed by using an Axon Multiclamp 700B Microelectrode Amplifier (Molecular Devices, USA) at room temperature (21–25 °C). Patch pipettes were fabricated from glass capillary tubes by PC-10 Puller (Narishige, Japan) with the resistance of 2~3 MΩ. Data acquisition and stimulation protocols were controlled by a Pentium III computer (Legend, Beijing, China) equipped with pCLAMP10.3 (Molecular Devices, USA). Capacitance transients were cancelled. Cells with a seal resistance (Rseal) below 1 GΩ were omitted. Series resistance (Rs) was compensated (80%) to minimize voltage errors, and cells with an uncompensated series resistance (Rs) above 10 MΩ were omitted. Leak subtraction was performed using P/4 protocol. Data were lowpassed at 10 kHz. The rate of solution exchange was studied using solutions with different KCl concentrations and found to be about 95% complete within 20 s. The holding potential was −100 mV. Unless stated specially, all the recordings were done with the pulse of +40 mV.

### Solutions and drugs

In the patch-clamp recordings, the standard bath solution channels was consisted of the following (in mM) NaCl 135, KCl 5, MgCl_2_ 1, CaCl_2_ 1.8, HEPES 10, glucose 10 (pH 7.4 titrated with NaOH). Pipette solutions for both Kv4.2 and Kv1.3 were composed of the following (in mM): KCl 130, MgCl_2_ 0.5, MgATP 2, EGTA 10, HEPES 10 (pH 7.3 titrated with KOH).

Synthetic BmP02 and its mutants (Figure S1) were purchased from TOP PEPTIDE, China, which were synthesized with a CEM microwave peptide synthesizer (Matthews, NC, USA) using the solid-phase method and cyclized by oxidative folding method. The secondary structures of wild-type BmP02 and the mutants were experimentally measured by Circular Dichroism (CD) spectroscopy (JASCO J-815, Japan). The NMR experiments were acquired on a Varian Unity Inova 600 spectrometer with cryo-probe equipped with three channels and pulse-field gradient.

The toxin was dissolved in the bath solution, supplemented with 1 mg/mL bovine serum albumin (BSA) in order to prevent adherence of the toxin to the vials and the perfusion apparatus. Application of 1 mg/mL BSA alone did not alter Kv1.3, Kv3.1a and KCa1.1 channel function. Unless otherwise stated, all reagents were purchased from Sigma.

### 3D Modeling

Utilizing the X-ray crystal structures of KcsA Potassium channel (PDB: 1BL8)^21^ as the templates, 3D model of Kv1.3 were generated using the Build homology models (MODELER) in Discovery Studio 4.0 (Accelrys, San Diego, CA, USA). The X-ray crystal structures of BmP02 (PDB: 1DU9)[12] and 3D model of Kv1.3 were used for docking analysis. The best Kv1.3-BmP02 complex was chosen according to the ZDock score.

CHARMm forcefield was added to the best Kv1.3-BmP02 complex. The “Standard Dynamics Cascade” was used to run the MD simulations. 1000 steps steepest descent minimization and 2000 steps adopted bassis NR were performed. Then the system was heated from 50 K to 300 K in 50 ps and keep 300 K for 500 ps in order to equilibration under an isothermal-isobaric (NPT). The conformations were saved every 2 ps. Finally, 5000 conformations were got in 10000 ps.

The energetic effect of each mutation on the binding affinity is calculated as the difference between the binding free energy in the mutated structures and the wild type protein. All interaction energy terms are calculated by CHARMm using a Generalized Born implicit solvent model and contain empirically scaled contributions of van der Waals and electrostatic interactions and a non-polar solvation energy term. The input structure of Kv1.3-BmP02 complex was obtained from the cluster analysis of the MD simulation trajectory.

### Data analysis

Data were analyzed by ClampFit 10.3 (Molecular Devices, USA) and Origin 8.5 (Northampton, Massachusetts, USA). Results of data analysis were expressed as mean ± S.E.M. and n represents the number of the cells examined. The Statistical significance was determined using the unpaired Student’s t-Test or one-way ANOVA, and an asterisk denotes P < 0.05 unless otherwise stated.

Dose-response curve was drawn according to the Hill equation I = I_m_/(1 + ([toxin]/EC_50_)n), where I_m_ is maximum of the current, and [toxin] is the concentration of sBmP02. EC_50_ (half-maximal effective concentration) and n denote the toxin concentration of half maximal effect.

Kv1.3 channel currents was elicited by the step pulses ranging from −100 to +60 mV for 200 ms with the increments of 10 mV (The holding potentials were held at −100 mV). For determining the voltage dependence of activation, the conductance was calculated using the formula: G(V) = I(V)/(V − ErK), where I(V) is the currents of Kv1.3 channel at the command voltage V, and ErK is the reversal potential. The conductance were normalized to the maximal value and the voltage dependence for activation of Kv1.3 channel fitted to a Boltzmann equation: G/Gmax = 1/[1 + exp(V-V1/2)/k], where V1/2 is the voltage for half-maximum activation and k is the slope factor.

## Additional Information

**How to cite this article**: Wu, B. *et al*. Mapping the Interaction Anatomy of BmP02 on Kv1.3 Channel. *Sci. Rep.* **6**, 29431; doi: 10.1038/srep29431 (2016).

## Supplementary Material

Supplementary Information

## Figures and Tables

**Table 1:** The bonds forming by the three Key residues to Kv1.3 channel vestibule.

Ligand residues	Receptor residue	Bond type	Distance (Å)
HIS9	D:HIS451	Hydrogen bond	2.0377
HIS9	D:SER426	Hydrogen bond	1.8243
LYS11	D:ASP449	Hydrogen bond	2.1291
LYS11	A:TYR447	Hydrogen bond	1.7427
LYS11	B:TYR447	Hydrogen bond	1.7719
LYS11	A:GLY448	Hydrogen bond	1.6409
LYS11	C:GLY448	Hydrogen bond	1.5288
LYS11	D:TYR447	Hydrogen bond	1.9719
LYS11	A:HIS451	Hydrogen bond	2.6921
LYS11	B:GLY450	Hydrogen bond	3.0127
LYS11	B:TYR447	Hydrogen bond	2.4364
LYS13	B:GLY450	Hydrogen bond	1.8004
LYS13	B:MET452	Hydrogen bond	1.5996
LYS13	B:GLY450	Hydrogen bond	2.6768
LYS13	A:ASP449	Salt bridge	2.2948

The distance of the contact was less than 5 Å.

## References

[b1] Cahalan M. D. & Chandy K. G. The functional network of ion channels in T lymphocytes. Immunological reviews 231, 59–87, 10.1111/j.1600-065X.2009.00816.x (2009). 19754890 PMC3133616

[b2] Feske S. Calcium signalling in lymphocyte activation and disease. Nature reviews. Immunology 7, 690–702, 10.1038/nri2152 (2007). 17703229

[b3] Beeton C. . Kv1.3 channels are a therapeutic target for T cell-mediated autoimmune diseases. Proceedings of the National Academy of Sciences of the United States of America 103, 17414–17419, 10.1073/pnas.0605136103 (2006). 17088564 PMC1859943

[b4] Rangaraju S., Chi V., Pennington M. W. & Chandy K. G. Kv1.3 potassium channels as a therapeutic target in multiple sclerosis. Expert opinion on therapeutic targets 13, 909–924, 10.1517/14728220903018957 (2009). 19538097

[b5] Fu X. X. . 18beta-Glycyrrhetinic acid potently inhibits Kv1.3 potassium channels and T cell activation in human Jurkat T cells. Journal of ethnopharmacology 148, 647–654, 10.1016/j.jep.2013.05.022 (2013). 23707333

[b6] Zhao N. . Acacetin blocks kv1.3 channels and inhibits human T cell activation. Cellular physiology and biochemistry: international journal of experimental cellular physiology, biochemistry, and pharmacology 34, 1359–1372, 10.1159/000366343 (2014). 25301362

[b7] Tytgat J. . A unified nomenclature for short-chain peptides isolated from scorpion venoms: alpha-KTx molecular subfamilies. Trends in pharmacological sciences 20, 444–447 (1999). 10542442 10.1016/s0165-6147(99)01398-x

[b8] Saucedo A. L. . New tricks of an old pattern: structural versatility of scorpion toxins with common cysteine spacing. The Journal of biological chemistry 287, 12321–12330, 10.1074/jbc.M111.329607 (2012). 22238341 PMC3320981

[b9] Banerjee A., Lee A., Campbell E. & Mackinnon R. Structure of a pore-blocking toxin in complex with a eukaryotic voltage-dependent K(+) channel. eLife 2, e00594, 10.7554/eLife.00594 (2013). 23705070 PMC3660741

[b10] Dauplais M. . On the convergent evolution of animal toxins. Conservation of a diad of functional residues in potassium channel-blocking toxins with unrelated structures. The Journal of biological chemistry 272, 4302–4309 (1997). 9020148 10.1074/jbc.272.7.4302

[b11] Tudor J. E., Pallaghy P. K., Pennington M. W. & Norton R. S. Solution structure of ShK toxin, a novel potassium channel inhibitor from a sea anemone. Nature structural biology 3, 317–320 (1996). 8599755 10.1038/nsb0496-317

[b12] Faure G., Xu H. & Saul F. A. Crystal structure of crotoxin reveals key residues involved in the stability and toxicity of this potent heterodimeric beta-neurotoxin. Journal of molecular biology 412, 176–191, 10.1016/j.jmb.2011.07.027 (2011). 21787789

[b13] Aguilar M. B. . Peptide sr11a from Conus spurius is a novel peptide blocker for Kv1 potassium channels. Peptides 31, 1287–1291, 10.1016/j.peptides.2010.04.007 (2010). 20403399

[b14] Pennington M. W. . An essential binding surface for ShK toxin interaction with rat brain potassium channels. Biochemistry 35, 16407–16411, 10.1021/bi962463g (1996). 8987971

[b15] Romi-Lebrun R. . Characterization of four toxins from Buthus martensi scorpion venom, which act on apamin-sensitive Ca^2+^-activated K^+^ channels. European journal of biochemistry/FEBS 245, 457–464 (1997). 9151979 10.1111/j.1432-1033.1997.00457.x

[b16] Xu Y. . Solution structure of BmP02, a new potassium channel blocker from the venom of the Chinese scorpion Buthus martensi Karsch. Biochemistry 39, 13669–13675 (2000). 11076505 10.1021/bi000860s

[b17] Tong Q. C., Zhang Y., Li D. P., Zhou Z. N. & Ji Y. H. The blocking effect of BmP02, one novel short-chain scorpion peptide on transient outward K(+) channel of adult rat ventricular myocyte. Regulatory peptides 90, 85–92 (2000). 10828497 10.1016/s0167-0115(00)00116-6

[b18] Zhu S. . Molecular cloning and sequencing of two ‘short chain’ and two ‘long chain’ K(+) channel-blocking peptides from the Chinese scorpion Buthus martensii Karsch. FEBS letters 457, 509–514 (1999). 10471839 10.1016/s0014-5793(99)01101-1

[b19] Mahjoubi-Boubaker B., Crest M., Khalifa R. B., El Ayeb M. & Kharrat R. Kbot1, a three disulfide bridges toxin from Buthus occitanus tunetanus venom highly active on both SK and Kv channels. Peptides 25, 637–645, 10.1016/j.peptides.2004.02.017 (2004). 15165720

[b20] Zhu L., Gao B., Luo L. & Zhu S. Two dyad-free Shaker-type K(+) channel blockers from scorpion venom. Toxicon: official journal of the International Society on Toxinology 59, 402–407, 10.1016/j.toxicon.2011.11.016 (2012). 22239942

[b21] Doyle D. A. . The structure of the potassium channel: molecular basis of K+ conduction and selectivity. Science 280, 69–77 (1998). 9525859 10.1126/science.280.5360.69

[b22] Lange A. . Toxin-induced conformational changes in a potassium channel revealed by solid-state NMR. Nature 440, 959–962, 10.1038/nature04649 (2006). 16612389

[b23] Zhu S. . Experimental conversion of a defensin into a neurotoxin: implications for origin of toxic function. Molecular biology and evolution 31, 546–559, 10.1093/molbev/msu038 (2014). 24425781

[b24] Rashid M. H. & Kuyucak S. Free energy simulations of binding of HsTx1 toxin to Kv1 potassium channels: the basis of Kv1.3/Kv1.1 selectivity. The journal of physical chemistry. B 118, 707–716, 10.1021/jp410950h (2014). 24397610

[b25] Rashid M. H. & Kuyucak S. Affinity and selectivity of ShK toxin for the Kv1 potassium channels from free energy simulations. The journal of physical chemistry. B 116, 4812–4822, 10.1021/jp300639x (2012). 22480371

[b26] Li Z. . Selective inhibition of CCR7(−) effector memory T cell activation by a novel peptide targeting Kv1.3 channel in a rat experimental autoimmune encephalomyelitis model. The Journal of biological chemistry 287, 29479–29494, 10.1074/jbc.M112.379594 (2012). 22761436 PMC3436147

