# Supplementary material for: Mapping the Interaction Anatomy of BmP02 on Kv1.3 Channel
Source: Sci Rep. 2016 Jul 11;6:29431. doi: 10.1038/srep29431 (PMC4941521; doi:10.1038/srep29431)
Supplement: Supplementary Information [file srep29431-s1.doc]

**Supplementary materials:**

**Mapping the Interaction Anatomy of BmP02 on Kv1.3 Channel**

Wu B^1^., Wu B.F^1^., Feng Y.J^1^., Tao J^2^*., Ji Y.H^1^*

**
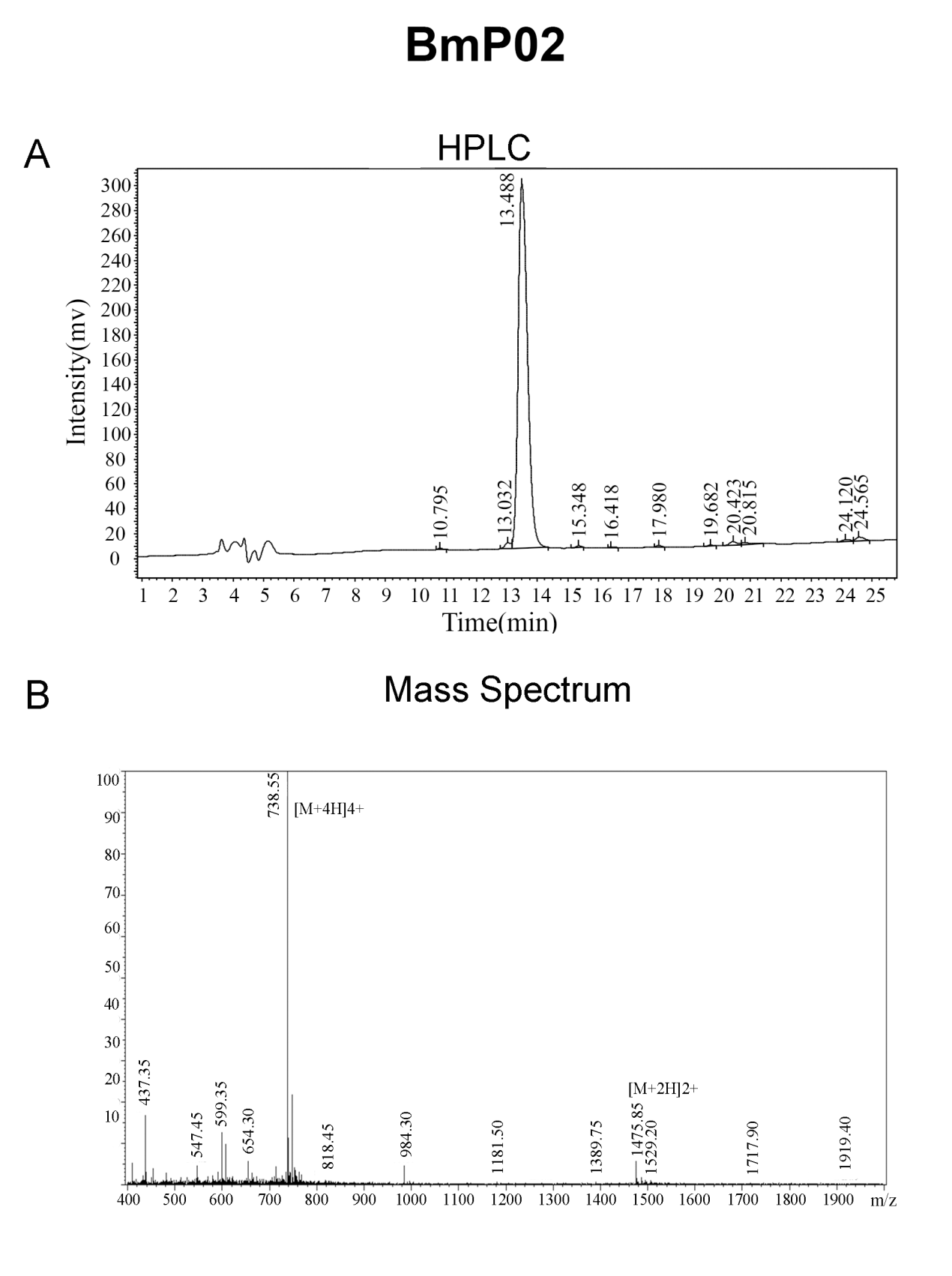
**


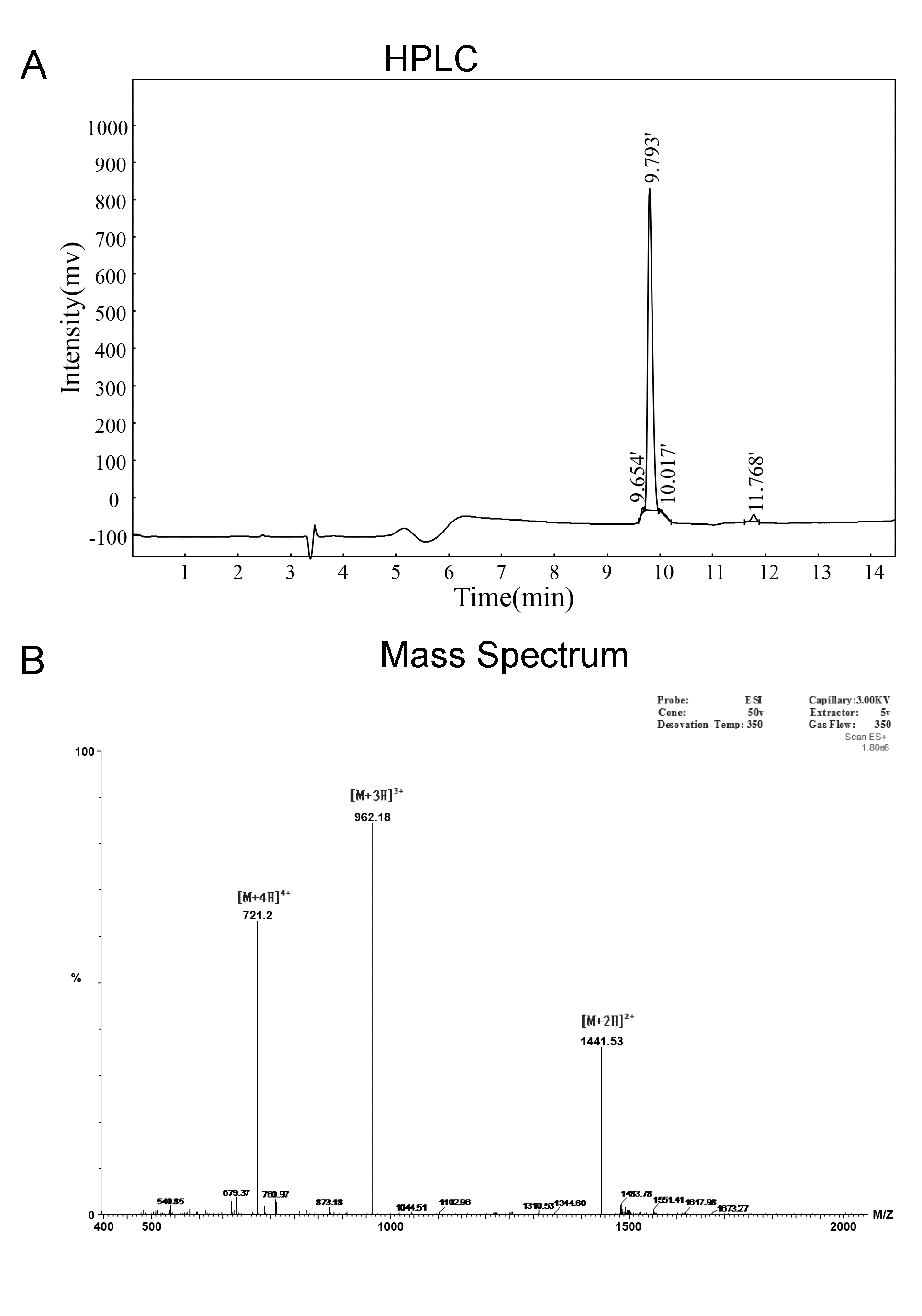


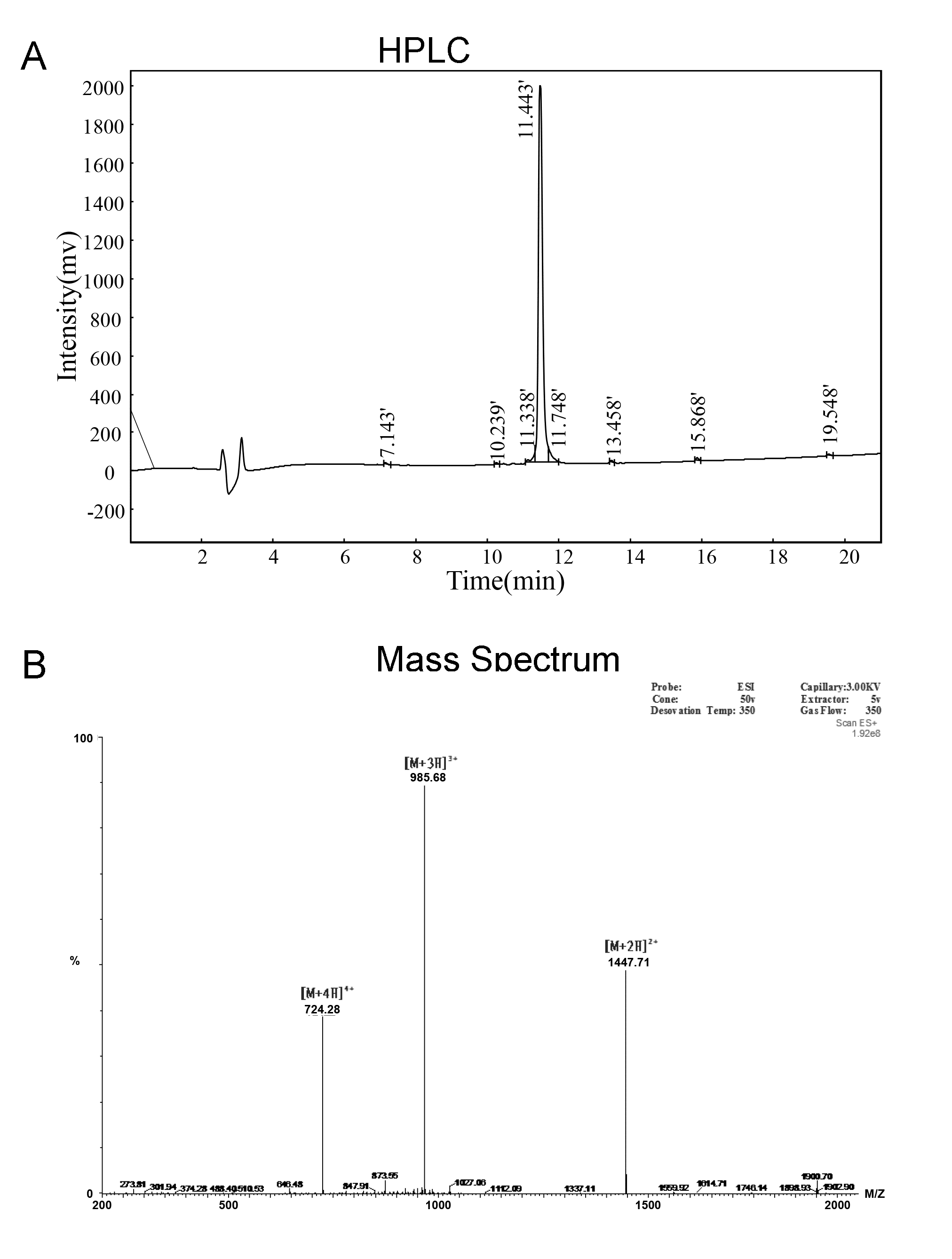

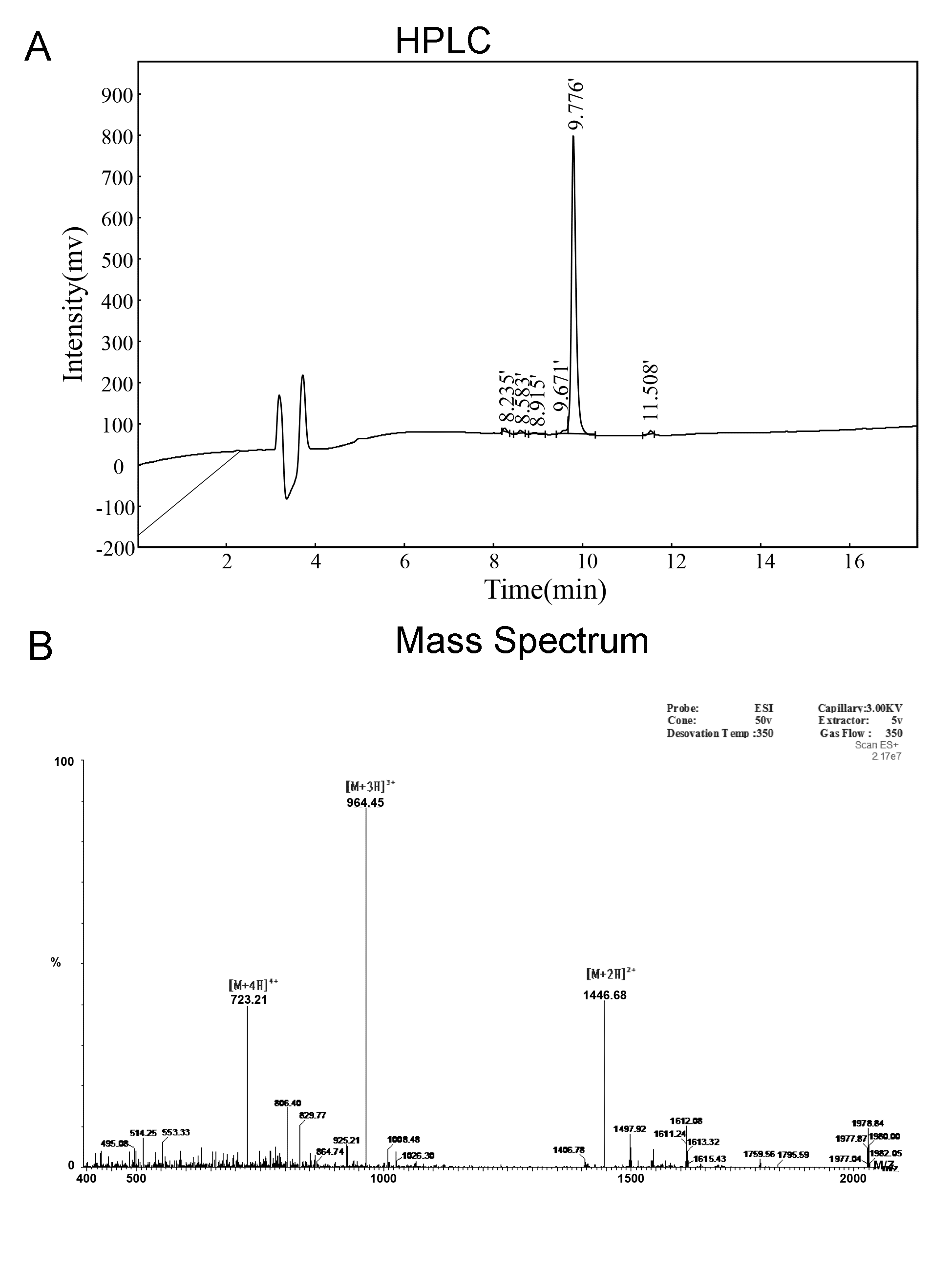


Figure S1-S4. Chemical synthesis and purification of synthetic BmP02 and H9A, K11A, K13A mutants. A. Purity of these peptides were determined by HPLC. B. Molecular weights of the peptide were determined by mass spectrum (shown in table s1).

| Name | Rank | Time (min) | Concentration (%) | Molecular weight |
| --- | --- | --- | --- | --- |
| BmP02 | 1 | 10.795 | 0.1758 | 2949.4 |
|  | 2 | 13.032 | 1.0112 |  |
|  | 3 | 13.488 | 95.6989 |  |
|  | 4 | 15.348 | 0.2137 |  |
|  | 5 | 16.418 | 0.0852 |  |
|  | 6 | 17.980 | 0.1757 |  |
|  | 7 | 19.682 | 0.1272 |  |
|  | 8 | 20.423 | 0.7366 |  |
|  | 9 | 20.815 | 0.5413 |  |
|  | 10 | 24.120 | 0.4065 |  |
|  | 11 | 24.565 | 0.8279 |  |
| H9A | 1 | 9.654 | 1.03 | 2883.33 |
|  | 2 | 9.793 | 96.05 |  |
|  | 3 | 10.017 | 0.598 |  |
|  | 4 | 11.768 | 2.32 |  |
| K11A | 1 | 7.143 | 0.1427 | 2892.3 |
|  | 2 | 10.239 | 0.2171 |  |
|  | 3 | 11.338 | 1.827 |  |
|  | 4 | 11.443 | 94.95 |  |
|  | 5 | 11.748 | 2.168 |  |
|  | 6 | 13.458 | 0.2185 |  |
|  | 7 | 15.868 | 0.3486 |  |
|  | 8 | 19.548 | 0.1302 |  |
| K13A | 1 | 8.235 | 0.8616 | 2892.3 |
|  | 2 | 8.583 | 0.9283 |  |
|  | 3 | 8.915 | 0.6279 |  |
|  | 4 | 9.671 | 2.132 |  |
|  | 5 | 9.776 | 94.4 |  |
|  | 6 | 11.508 | 1.056 |  |

Table S1. Purity and molecular weight of the BmP02 mutants.


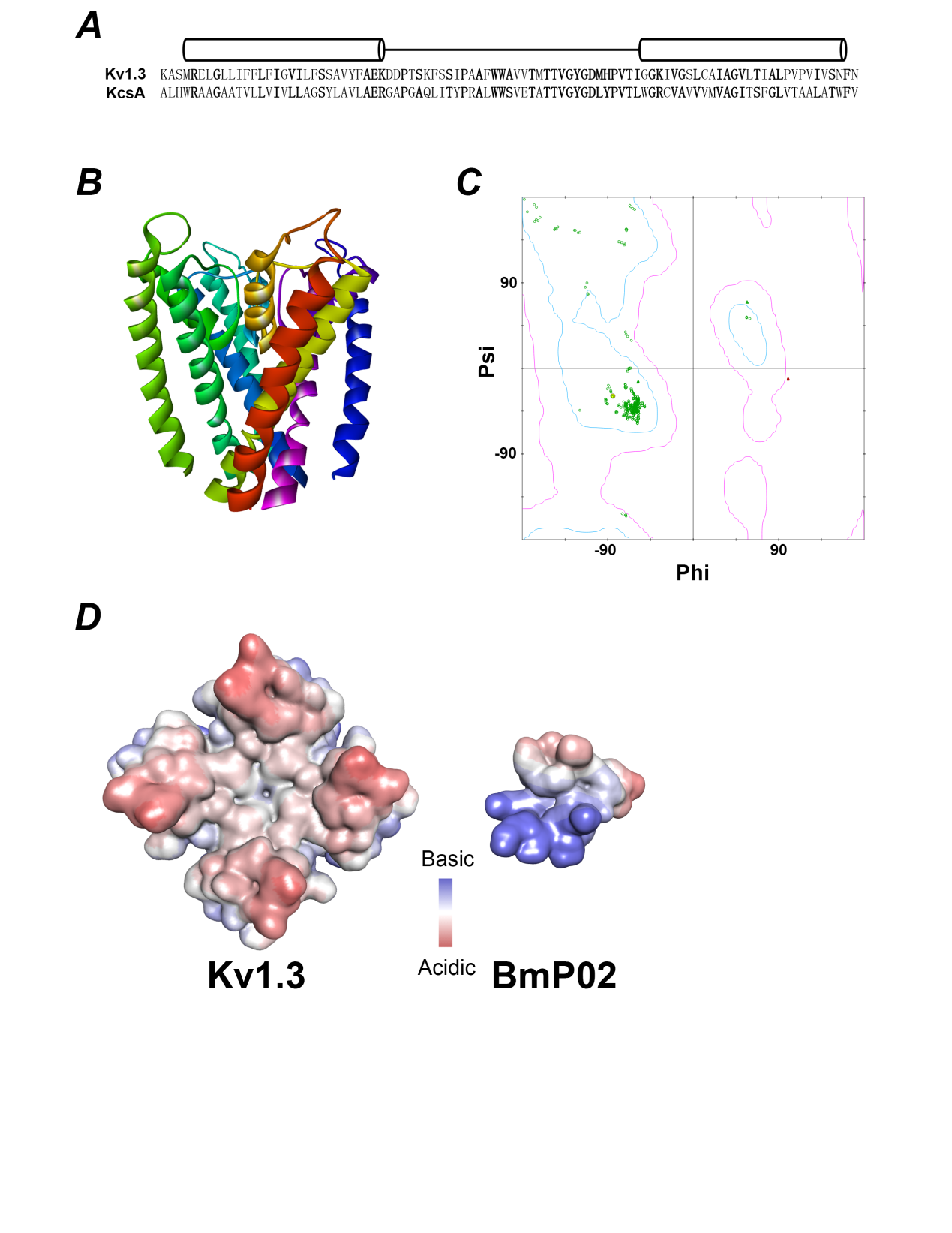


Figure S5. Homology Modeling of Kv1.3 channels. A. Sequence alignment of Kv1.3 and the template KcsA (PDB: 1BL8). The sequence similarity is 52.1%. B. Ribbon diagram of Kv1.3 Channel ionic pore from Homology Modeling. C. the Ramachandran plot of Kv1.3 Channel ionic pore. Over 99% residues located in the Allowed Area. D. The pKa surface of Kv1.3 and BmP02.


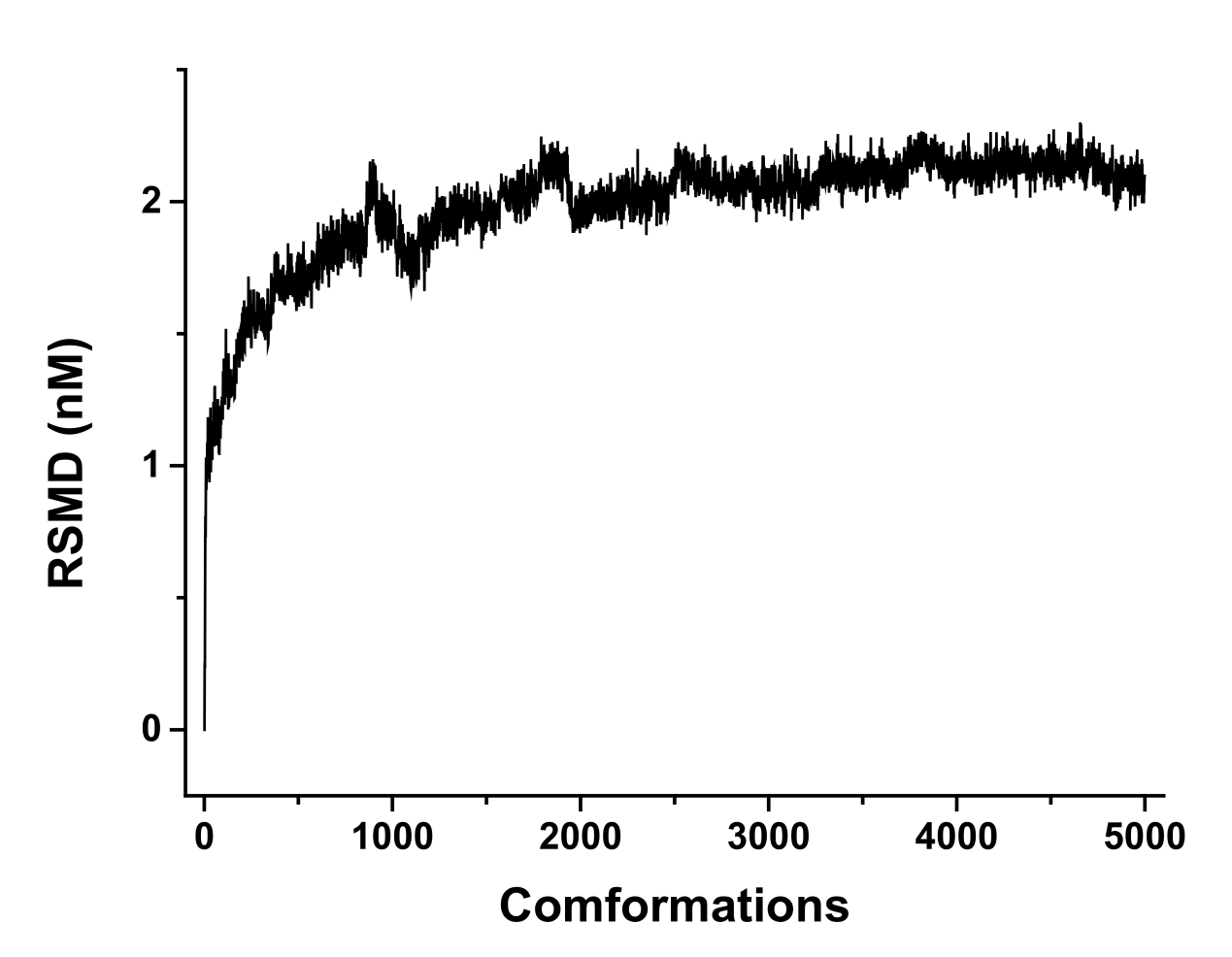


Figure S6. The analysis of Root Mean Square Deviation of Kv1.3 Channel-BmP02 complex during molecular dynamics simulation.


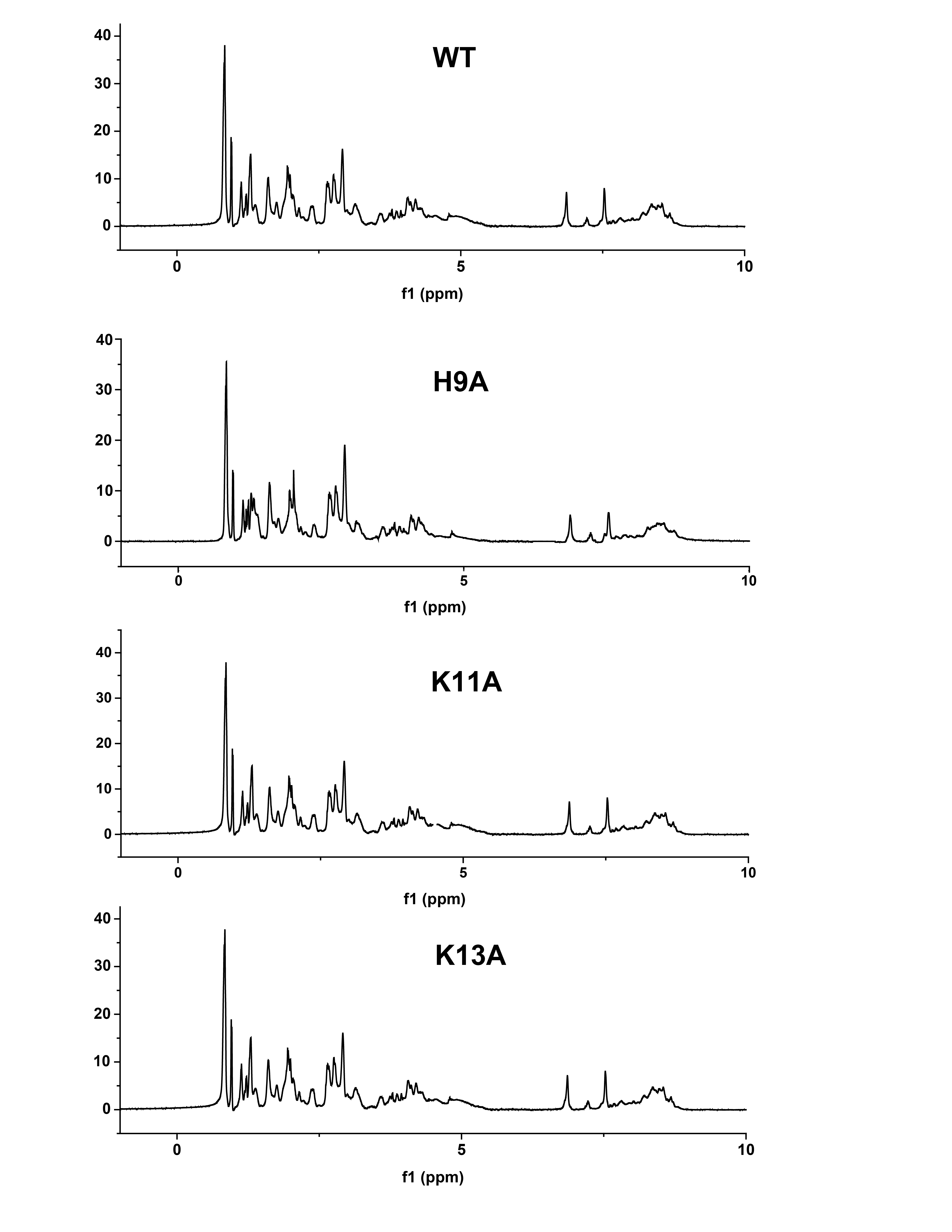


Figure S7. The ^1^H NMR of the wild type and the three mutants BmP02, respectively.


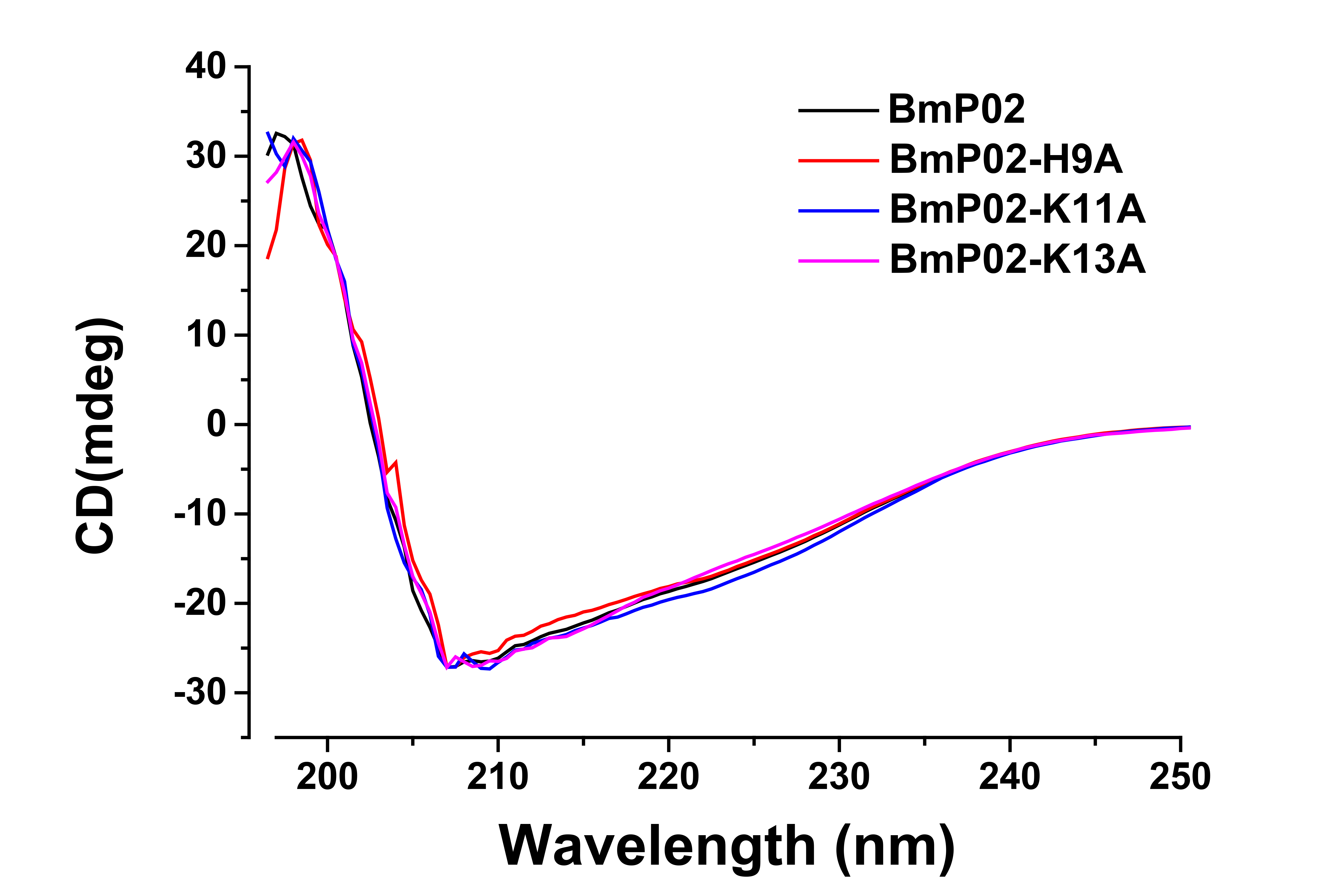


Figure S8. The CD spectra of the wild type and the three mutants BmP02.

| Mutants | Primers |
| --- | --- |
| H451V | S:5’-GGTGATATGGTCCCAGTGACCATAGGAGG |
|  | A:5’-GGTCACTGGGACCATATCACCATAACCAA |
| D433A | S:5’-AGTATCCCGGCAGCCTTCTGGTGGGCAGT |
|  | A:5’-CCAGAAGGCTGCCGGGATACTGTTAAAAC |
| D421A | S:5’-CTGAGGCAGCAGACCCTTCTTCGGGTTT |
|  | A:5’-GAAGGGTCTGCTGCCTCAGCAAAGTAGA |
| D422A | S:5’-GAGGCAGACGCACCTTCTTCGGGTTTTAA |
|  | A:5’-GAAGAAGGTGCGTCTGCCTCAGCAAAGT |

Table S2. The primers used in the construction of mutants. The mutated sites are underlined. S: sense, A: anti-sense.
